# Controllable Music Playlist Generation Based on Knowledge Graph and Reinforcement Learning †

**DOI:** 10.3390/s22103722

**Published:** 2022-05-13

**Authors:** Keigo Sakurai, Ren Togo, Takahiro Ogawa, Miki Haseyama

**Affiliations:** 1 Graduate School of Information Science and Technology, Hokkaido University, N-14, W-9, Kita-ku, Sapporo 060-0814, Hokkaido, Japan; 2 Faculty of Information Science and Technology, Hokkaido University, N-14, W-9, Kita-ku, Sapporo 060-0814, Hokkaido, Japan; togo@lmd.ist.hokudai.ac.jp (R.T.); ogawa@lmd.ist.hokudai.ac.jp (T.O.); miki@ist.hokudai.ac.jp (M.H.)

**Keywords:** music playlist generation, knowledge graph, reinforcement learning, multimedia techniques, music recommendation, preference sensing

## Abstract

In this study, we propose a novel music playlist generation method based on a knowledge graph and reinforcement learning. The development of music streaming platforms has transformed the social dynamics of music consumption and paved a new way of accessing and listening to music. The playlist generation is one of the most important multimedia techniques, which aims to recommend music tracks by sensing the vast amount of musical data and the users’ listening histories from music streaming services. Conventional playlist generation methods have difficulty capturing the target users’ long-term preferences. To overcome the difficulty, we use a reinforcement learning algorithm that can consider the target users’ long-term preferences. Furthermore, we introduce the following two new ideas: using the informative knowledge graph data to promote efficient optimization through reinforcement learning, and setting the flexible reward function that target users can design the parameters of itself to guide target users to new types of music tracks. We confirm the effectiveness of the proposed method by verifying the prediction performance based on listening history and the guidance performance to music tracks that can satisfy the target user’s unique preference.

## 1. Introduction

In our daily lives, we are constantly exposed to various situations that change our mental states. With the global acceleration of digital technologies, these changes have become larger and more complex [1]. Digital devices have driven innovation in society at an unprecedented speed, and new technologies are being created daily. It is important to have a relaxing and refreshing environment to maintain one’s mental state and motivation in this new era. Music is one of the primary ways to create such an environment. Music can lead to various changes in our mental states, i.e., creating positive as well as negative emotions [2,3].

The development of music streaming platforms, such as Spotify (https://www.spotify.com, accessed on 12 May 2022) and YouTube Music (https://music.youtube.com/, accessed on 12 May 2022) has transformed the social dynamics of music consumption and paved a new way to access and listen to music [4,5,6]. Consumers can access millions of music tracks on music streaming platforms at any time and from any location using mobile devices, such as smartphones and tablets [7]. This creates an interesting challenge for users to find their favorite music tracks among the overwhelming variety of music tracks on music streaming platforms [8].

Music streaming platforms provide various services and tools to their users, with playlist generation and recommendations being the most common [9]. According to a Spotify announcement in 2017, there are more than 4 billion playlists available [10]. Furthermore, according to a study conducted by the Music Business Association in 2016 [11], listeners in the United States spend 31% of their music listening time on playlists, which is longer than the time spent listening to albums. These reports also show the growing importance of playlists as a mode of music consumption. Generally, manual construction of playlists by users can be a comparably complicated and time-consuming task [12,13,14]. Multimedia techniques, including automatic playlist generation by sensing the vast amount of musical data and the users’ listening histories, contribute to releasing users from the effort involved in the process [15,16,17].

Conventionally, various music playlist generation methods have been proposed, such as the classical matrix factorization-based methods [18,19,20] and the recurrent neural network-based methods [21,22]. These methods have achieved high accuracy in predicting users’ short-term preferences for music tracks. However, almost all previous studies did not focus on predicting target users’ long-term preferences. Music playlist generation is a task that presents multiple music tracks as a list, unlike ordinary music recommendations, which predict and provide a single music track [23,24]. It is difficult for conventional methods based on short-term predictions to generate a playlist where all music tracks are preferred by users. We consider that all music tracks in a generated playlist should contribute to increasing user satisfaction.

Recent remarkable developments in reinforcement learning provide promising solutions to the problem by maximizing long-term performance [25,26]. Reinforcement learning (RL) is a method used for determining the optimal behavior that maximizes the reward in a single episode based on a Markov decision process (MDP) [27,28]. The goal of the RL-based playlist generation method is to select and list more music tracks that can satisfy users in a single episode by treating the length of the playlist as an episode. The reward function based on users’ listening histories and feedback from recommendation results has been used in conventional RL-based playlist generation methods [29,30]. These methods are designed to provide users with a pleasant playlist by learning the policy function to maximize the values of the reward function.

The following two aspects of the above trials are yet to be resolved: predicting users’ preferences based on listening histories and guiding users to new preferences. The problem in predicting users’ preferences is that learning the policy function directly toward optimization objectives from users’ listening histories, which are probably sparse or limited, is difficult [25,31]. Exploration and exploitation are the two basic learning processes in RL. The exploration to grasp users’ preferences may take a huge amount of time and successful optimization may not be guaranteed because listening histories are sparse. However, because the reward functions are based only on users’ listening histories and feedback from the recommendation results, it is possible to end up with echo chambers and filter bubbles when guiding users to new preferences [32,33]. It is not the primary purpose of a music playlist generation to extremely narrow down the range of users’ interests by exploiting their listening histories. Therefore, it is necessary to guide users to new types of music tracks beyond the scope of users’ past preferences using not only the past listening histories but also sensing explicit inputs from target users.

To solve these problems, this study presents a novel controllable music playlist generation method based on a knowledge graph and RL. In the proposed method, we newly introduce the following two ideas. First, to promote efficient optimization through RL, we use informative knowledge graph (KG) data. KG is a directed graph that can represent higher-order relationships among nodes [34,35]. The advantage of KG is that it is possible to compute informative features considering the relevance of rich metadata based on embedding methods [36]. The proposed method achieves effective optimization and solves the sparsity problem using the state representation based on music features obtained from KG in addition to users’ listening histories. Second, we create a flexible reward function that target users can design to guide target users to new types of music tracks. The reward function consists of indices to reflect users’ specific preferences for popularity, novelty, and acoustic feature similarity. Target users are directed to new types of music tracks based on the index they specify by manipulating the weight parameters of each index. We use the policy gradient, one of the RL algorithms, to determine the agent’s policy for generating playlists based on the states and rewards designed above. Finally, we generate music playlists for target users based on the obtained agent’s policy. Our contribution is the accurate prediction of target users’ preferences and the introduction of new types of music tracks to target users.

The rest of this paper is organized as follows. In Section 2, we present related works on music playlist generation, such as RL-based and KG-based recommendations. In Section 3, we provide preliminary information to support the understanding of our method. Section 4 explains the proposed music playlist recommendation method. Section 5 presents the experimental results using a real-world music playlist dataset. Finally, we conclude our study in Section 6.

## 2. Related Works

In this section, we present related works on a music playlist generation and KG-based recommendations to clarify the novelty and contribution of our study.

### 2.1. Music Playlist Generation

A music playlist generation problem can be considered a special case of music recommendation problems [37]. The order and characteristics of music tracks affect the quality of the playlist, which is a unique feature of music playlist creation. A previous study used machine learning techniques that consider a time series to tackle the problem of a music playlist generation [21,22,38,39,40,41]. Choi et al. used a recurrent neural network (RNN) to generate a music playlist that focused on the qualities of track transitions [21]. Monti et al. used an ensemble strategy of RNNs based on pre-trained embeddings representing albums, titles, etc. [38]. Irene et al. predicted users’ future preferences by analyzing playlists created manually based on RNN and a convolutional neural network (CNN) [22]. However, it is difficult to capture users’ long-term preferences because these approaches are trained to acquire sequence characteristics of a few steps.

Recently, RL-based music playlist generation methods have been studied to consider users’ long-term preferences. An important factor that influences the performance of RL-based playlist generation is the reward function. Hu et al. attempted to improve the performance of playlist generation by introducing user feedback on recommendation results into the reward function [30]. However, this approach may lead to a feedback loop that narrows the range of users’ interests. Shih et al. introduced novelty and popularity-based indices into the reward function and generated playlists containing many new and famous music tracks [42]. Our previous study focused on the “smooth track transition,” and we introduced indices based on acoustic feature similarity to the reward function [3,17]. However, there is no consensus in conventional RL-based playlist generation studies as to which indices of the reward function are better. To address this problem, we use an approach that allows users to create playlists they desire by manipulating the parameters of the reward function themselves.

### 2.2. Knowledge Graph-Based Recommendation

The recent development of KG in the field of recommender systems has been remarkable [43,44,45,46]. The approach of these KG-based recommendation studies uses the rich relation information of KG for improving the explainability and accuracy of recommender systems. Furthermore, higher performance can be achieved by combining KG and RL [36,47,48,49,50,51]. Xian et al. attempted KG reasoning for recommendation using the soft reward function based on KG embeddings [36]. Song et al. used a hard reward function based on user interaction information in addition to a soft reward function to improve recommendation accuracy [48]. Furthermore, our previous study introduced a soft reward function based on KG using acoustic features into the music recommendation algorithm [49]. However, it is unclear whether the KG is involved in the proper training of RL because these methods apply KG-based features only for the reward function.

Some researchers in the field of sequential recommendation have focused on how to use KG. Wang et al. have introduced KG embedding features into the state representation and the reward function of RL to accelerate the training of proper RL [25]. Based on this approach, we introduce music features, such as higher-order relation information obtained from the KG, into the music playlist generation algorithm. To the best of our knowledge, it is the first time that KG has been used in music playlist generation methods.

## 3. Preliminary

In this section, we present the preliminary to clarify our playlist generation task, especially the concept of RL. First, we introduce the notations in our task to make the process of playlist generation clear in advance. Furthermore, we explain the definition of our playlist generation task, and a MDP, which is a key concept of RL.

**Notation**
  A scenario of a music playlist generation consists of a target user’s listening history and his/her explicit inputs. Let U denote a set of users and M denote a set of music tracks in a database. For each target user $u_{v}\;(v=1,2,...,N_{u};\;N_{u}$ being the number of target users) ∈ U, we set $l_{1:N^{u_{v}}}^{u_{v}}=l_{1}^{u_{v}}\to l_{2}^{u_{v}}\to \cdots \to l_{N^{u_{v}}}^{u_{v}}$($N^{u_{v}}$ being the number of music tracks in the listening history of a target user $u_{v}$) to denote the listening history of the target user $u_{v}$. Specifically, $l_{1}^{u_{v}}$ denotes the music track that the target user $u_{v}$ listened to first. In addition to the listening history of a target user, our model uses the target user’s explicit inputs γ for the parameter of the reward function in RL. Furthermore, a KG G is provided for the task of music playlist generation, where each record is a triplet consisting of two entities and their relationships. The set of music tracks M can be arranged in the KG. Based on the KG, we can obtain associated knowledge information of music tracks, e.g., an artist of a music track or genres of an artist.
**Task definition**
  We used RL to generate music playlists. Based on the above notations, our model generates a music playlist and recommends it to a target user. The main advantage of our model is that it can predict the target user preferences based on the user’s listening history and guide the target user to the new types of music tracks based on the user’s explicit inputs.
**Markov decision process**
  MDP is an important principle of RL. First, we briefly introduce the MDP. Generally, the MDP can be described by a quintuple 〈S, A, T, R, π〉:
**State** S

  S denotes a set of states, and each s ∈ S represents the information state of an agent in the environment.
**Action** A

  A denotes a set of actions, and each a ∈ A denotes the actions that the agent can take with respect to the environment.
**Transition function** T

  T denotes a transition function for updating the state according to the action and current state, i.e., $s_{t+1}$ = T($s_{t}$, $a_{t}$).
**Reward function** R

  R denotes a reward function, e.g., if the agent performs $a_{t}$ in state $s_{t}$, it gives an immediate reward R($s_{t}$, $a_{t}$).
**Policy** π

  π denotes the agent’s action policy. Generally, it is modeled using a probability distribution over possible actions.
Based on the above five definitions, we obtained the optimal policy through repeated trial and error.

## 4. Proposed Method

**Figure 1:**
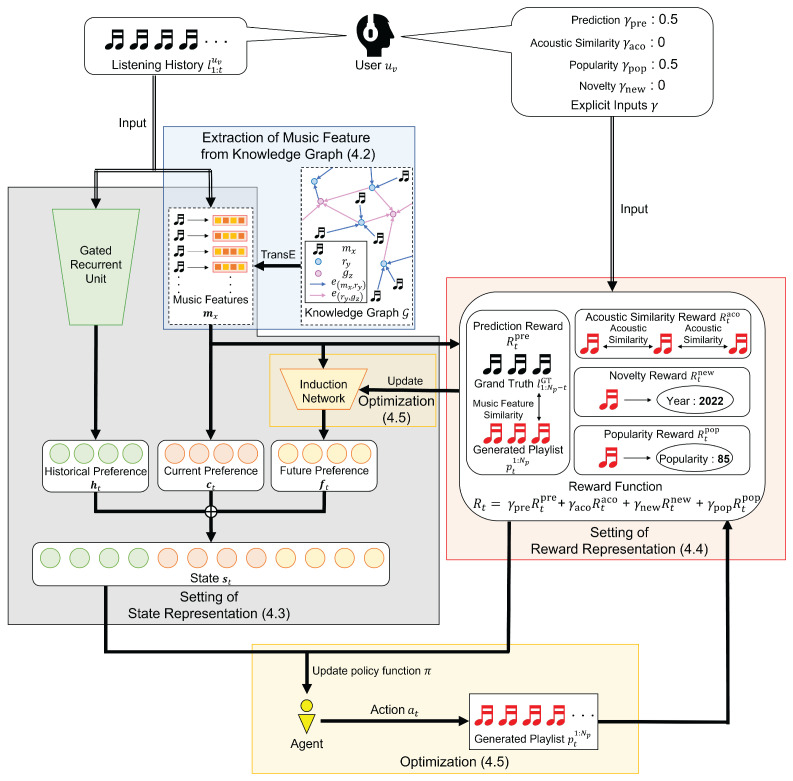
The overview of the proposed method. Based on the target user’s listening history $l_{1:t}^{u_{v}}$ and his/her explicit inputs γ, our model learns the policy function π for the agent that generates the playlist $p_{t}^{1:N_{p}}$ according to the MDP.

In this section, we introduce the proposed controllable music playlist generation method in detail. The overview of the proposed method is shown in Figure 1. Our method generates music playlists using the RL algorithm based on the target user’s listening history and their explicit inputs.

### 4.1. Formulation of Markov Decision Process

First, we use MDP to obtain the optimal policy and generate sophisticated playlists. In the MDP situation, the agent interacts with the environment at each time step and observes its state. In our task, the state of the environment contains the target user’s listening history and KG information describing the higher-order relationships of music tracks in the database. The state at step t is defined as follows:

(1) $$s_{t}=\left[l_{1:t}^{u_{v}},G\right],$$

where $l_{1:t}^{u_{v}}$ (1 ≤ t ≤ $N^{u_{v}}$) denotes the music track sequence in the listening history $l_{1:N^{u_{v}}}^{u_{v}}$ and G denotes the KG. Here, the initial state $s_{0}$ is defined as follows:

(2) $$s_{0}=\left[\emptyset ,G\right].$$

Furthermore, the agent takes an action $a_{t}$ ∈ A, which selects music tracks from the music track set M and generates a playlist $p_{t}^{1:N_{p}}$ ($N_{p}$ being the number of music tracks in the playlist) for the target user $u_{v}$ at step t. Specifically, $p_{t}^{1}$ is the first music track in the generated playlist at step t. The agent’s actions are determined on the basis of its policy π($a_{t}|s_{t}$). In our model, we define the policy π($a_{t}|s_{t}$) as a softmax function that outputs a probability of each music track. The policy π($a_{t}|s_{t}$) is defined as follows:

(3) $$\begin{array}{c} \pi \left(a_{t}|s_{t}\right)=\frac{\exp\{p_{t}Ws_{t}\}}{\sum_{m^{\prime }\in M}\exp\left\{m^{\prime }Ws_{t}\right\}}, \end{array}$$

(4) $$\begin{array}{c} p_{t}=\sum_{i=1}^{N_{p}}\frac{p_{t}^{i}}{N_{p}}, \end{array}$$

where $m^{\prime }$ and $p_{t}^{i}$ denote the music features of $m^{\prime }$ and $p_{t}^{i}$ obtained in Section 4.2, respectively, W denotes the parameters of the policy function, and $s_{t}$ is the embedding feature of state $s_{t}$.

According to the action, the agent obtains a reward $r_{t+1}$. The reward is designed based on the user’s listening history and explicit inputs from the target user. Furthermore, the transition function $T(s_{t},a_{t})$ representing the alteration to the next state can be defined as follows:

(5) $$\begin{array}{cc} T(s_{t},a_{t})= & T(\left[l_{1:t}^{u_{v}},G\right],p_{t}^{1:N_{p}}) \end{array}$$

(6) $$\begin{array}{cc} = & s_{t+1}. \end{array}$$

The new state $s_{t+1}$ is defined as [$l_{1:t+1}^{u_{v}}$, G] and associated with an embedding feature $s_{t+1}$. Based on the above formulation of MDP, we generate a sophisticated playlist by gradually updating the policy from the listening history and the explicit inputs.

### 4.2. Extraction of Music Feature from Knowledge Graph

In this subsection, we explain how to calculate music features using a KG. KG is a directed graph that can represent higher-order relationships among nodes. In the proposed method, we construct the KG using music tracks and their metadata. The KG G having a node set N and an edge set E is defined as G = {($n_{\mathrm{head}},e,n_{\mathrm{tail}})|n_{\mathrm{head}},n_{\mathrm{tail}}\in N,e\in E$}, where $n_{\mathrm{head}}$ and $n_{\mathrm{tail}}$ are a head node and a tail node, respectively, and e is an edge connecting $n_{\mathrm{head}}$ with $n_{\mathrm{tail}}$. In the proposed method, these nodes consist of music tracks $m_{x}\;(x=1,2,...,N_{m};\;N_{m}$ denotes the number of music tracks), artists $r_{y}\;(y=1,2,...,N_{r};\;N_{r}$ denote the number of artists) and genres $g_{z}\;(z=1,2,...,N_{g};\;N_{g}$ denote the number of genres of music tracks). Furthermore, we define “the directed edge $e_{\left(r_{y},m_{x}\right)}$ connecting music track $m_{x}$ to artist $r_{y}$ that created the music track $m_{x}$” and “the directed edge $e_{\left(m_{x},g_{z}\right)}$ connecting music track $m_{x}$ to genres $g_{z}$ that artist $r_{y}$ belongs to.” By using the directed graph, we can understand the hierarchical relationships of the heterogeneous entities.

Furthermore, we use TransE [52], which is a typical KG embedding method, to design KG G and calculate the embedding features of each node and edge. In TransE, features of nodes and edges are calculated to satisfy the following equation:

(7) $$n_{\mathrm{head}}+e=n_{\mathrm{tail}},$$

where $n_{\mathrm{head}}$, e and $n_{\mathrm{tail}}$ are embedding features of $n_{\mathrm{head}}$, e and $n_{\mathrm{tail}}$, respectively. Here, we define the embedding features of $m_{x}$, $r_{y}$, $g_{z}$, $e_{\left(r_{y},m_{x}\right)}$ and $e_{\left(m_{x},g_{z}\right)}$ as $m_{x}$, $r_{y}$, $g_{z}$, $e_{\left(r_{y},m_{x}\right)}$ and $e_{\left(m_{x},g_{z}\right)}$, respectively. Furthermore, we denote the embedding features $m_{x}$ as the music features of each music track. In this way, we can obtain music features that can represent higher-order relationships between music tracks and their metadata.

### 4.3. Setting of State Representation

Modeling the appropriate state representation is an important feature of the RL algorithm. We incorporate knowledge information from KG to learn a good state representation and set up the following three types of state representations: past preference-based representation, current preference-based representation, and future preference-based representation. This allows us to construct an RL learning algorithm that understands user preferences using rich information from the KG.

#### 4.3.1. Historical Preference State Representation

First, we used the gated recurrent unit (GRU) [53], which is a standard recurrent neural network (RNN) model, for encoding the target user’s listening history. The objective of the representation is to capture the characteristics of the user’s historical listening preferences, and it does not use information obtained from the KG. The historical preference-based state representation $h_{t}$ is defined as follows:

(8) $$h_{t}=\mathrm{GRU}\left(h_{t-1},l_{1:t}^{u_{v}};\Phi_{\mathrm{gru}}\right)$$

where $\Phi_{\mathrm{gru}}$ denotes all related parameters of the GRU.

#### 4.3.2. Current Preference State Representation

Second, we used the target user’s listening history and the music features calculated in Section 4.2. The objective of the representation is to obtain the characteristics of the music track content preferred by the users from the rich information of the KG. Based on the literature [25], we use a mean pooling strategy to aggregate KG features of music tracks played by the target user. The current preference-based state representation is defined as follows:

(9) $$c_{t}=\sum_{j=1}^{t}\frac{l_{j}^{u_{v}}}{t},$$

where $l_{j}^{u_{v}}$ denotes the music feature of $l_{j}^{u_{v}}$. Note that the above equation does not consider temporal information or attention mechanisms because there is no significant performance improvement over the simple representation described above.

#### 4.3.3. Future Preference State Representation

Third, we introduce the future preference to grasp the interest evolution of the target user at future time steps. We use an induction network to predict future preferences based on current preferences as the key point to generating a sophisticated music playlist. In our model, the induction network is developed using a multi-layer perceptron (MLP). We predict a future preference representation using the current preference-based representation $c_{t}$ as an input. The future preference-based state representation is defined as follows:

(10) $$f_{t}=\mathrm{IDN}\left(c_{t};\Phi_{\mathrm{idn}}\right),$$

where IDN (·) denotes the induction network constructed based on the MLP, $\Phi_{\mathrm{idn}}$ denotes the parameters of the induction network. We assume that the target user’s preference for the content of music tracks should not change much over successive time steps. Therefore, our goal is to predict the future preference based on the target user’s current preference. Learning the future preference from KG is useful for RL-based algorithms because it is an important point in developing a rational and profitable search. By using the MLP, the induction network is expected to grasp the growth of the target user’s preferences.

#### 4.3.4. Final State Representation

From the above discussion, we present the final state representation. For a final state $s_{t}$, we defined its representation $s_{t}$ as the concatenation of three representation embeddings: The final state representation is defined as follows:

(11) $$s_{t}=h_{t}⊕c_{t}⊕f_{t},$$

where ⊕ denotes the concatenation operator, $h_{t}$, $c_{t}$ and $f_{t}$ are obtained in Section 4.3.1, Section 4.3.2 and Section 4.3.3, respectively. Our model can generate music playlists containing music tracks predicted to be preferred by the target user by capturing the past, current, and future preferences of the target user.

### 4.4. Setting of Reward Function

In the subsection, we set the reward function. Our model’s main goals are to predict target users’ preferences based on their listening histories and to guide the target user to new types of music tracks based on their explicit inputs. To achieve both purposes, we introduce two types of rewards. The first is a prediction reward based on the similarity of the generated playlist and the ground truth listening history. The other is the guiding reward reflecting the target user’s specific preference for acoustic features similarity, year, and popularity of music tracks. Our model can generate playlists personalized to each user’s specific preferences by allowing the target user to control the balance of those rewards based on explicit inputs. In the following, we describe each reward.

#### 4.4.1. Prediction Reward

In the prediction reward, we consider measuring the quality of music features of the generated playlist. Exact matching between the ground truth (GT) listening history and the generated playlist is an effective solution for predicting music tracks. However, training for exact matching may be difficult because GT listening histories are sparse for the entire database. To avoid the sparse problem and promote efficient training, one of the rational solutions is to exploit the similarity of music features obtained in Section 4.2. The similarity of music features is not sparse, and music features representing higher-order relationships of metadata of the music tracks lead to approximate matching. Therefore, we introduce cosine similarity between music features of GT listening history and the generated playlist into the prediction reward, which solves the sparse problem and facilitates efficient training. Given the GT listening history and the generated playlist, namely $l_{1:N_{p}-t}^{\mathrm{GT}}$ and $p_{t}^{1:N_{p}}$, we use the simple average method to aggregate the music feature of music tracks, denoted by $c_{t}^{\mathrm{GT}}$ and $p_{t}^{1:N_{p}}$, respectively. In this way, the prediction reward $R_{t}^{\mathrm{pre}}$ is defined as follows:

(12) $$R_{t}^{\mathrm{pre}}=\frac{c_{t}^{\mathrm{GT}}\cdot {p_{t}^{1:N_{p}}}^{⊤}}{\left|\right|c_{t}^{\mathrm{GT}}\left|\right|\cdot \left|\right|{p_{t}^{1:N_{p}}}^{⊤}\left|\right|}.$$

The prediction reward $R_{t}^{\mathrm{pre}}$ is also used to train the induction network to update future state $f_{t}$ because it has a significant impact on the prediction performance of music tracks. The training of the induction network is discussed in detail in Section 4.5.

#### 4.4.2. Guiding Reward

The guiding reward aims to direct users to new types of music tracks that can satisfy their unique preferences. It consists of the following three indices: acoustic similarity, popularity, and novelty rewards. We assume that the target user’s unique preferences change over time and under different circumstances and that they cannot be recognized solely based on their listening history. To capture the user’s temporal unique preference, we receive reward function parameters from the user as explicit inputs and use them for balancing each reward.

**Acoustic Similarity Reward**
  Conventionally, playlists with smooth track transitions are effective in increasing users’ satisfaction [17,54]. We design a reward based on the similarity of acoustic features of music tracks for users who prefer playlists with highly smooth track transitions. Specifically, the acoustic similarity reward $R_{t}^{\mathrm{aco}}$ is calculated using the cosine similarity of the acoustic feature of the i-th and “i+1”-th music tracks in the generated playlist and defined as follows:
  (13) $$R_{t}^{\mathrm{aco}}=\frac{1}{N_{p}}\sum_{i=0}^{N_{p}-1}\frac{p_{t,\mathrm{aco}}^{i}\cdot {p_{t,\mathrm{aco}}^{i+1}}^{⊤}}{\left|\right|p_{t,\mathrm{aco}}^{i}\left|\right|\cdot \left|\right|{p_{t,\mathrm{aco}}^{i+1}}^{⊤}\left|\right|},$$
  where $p_{t,\mathrm{aco}}^{i}$ denotes the acoustic feature of the music track $p_{t}^{i}$.
**Popularity Reward**
  We assume that users who are unfamiliar with music or who are meek often listen to music based on its popularity. Many music streaming services have gained popularity by recommending popular music tracks to users who do not know what types of music they like. This means that popular music can attract users’ attention. To accommodate users who want to listen to popular songs, we use the value of popularity obtained from the Spotify API (https://developer.spotify.com/documentation/web-api/, accessed on 12 May 2022) in the popularity reward $R_{t}^{\mathrm{pop}}$, which is defined as follows:
  (14) $$R_{t}^{\mathrm{pop}}=\frac{1}{N_{p}}\sum_{i=1}^{N_{p}}\frac{\mathrm{Popularity}(p_{t}^{i})}{\mathrm{Popularity}(p_{\mathrm{highest}})},$$
  where Popularity(·) denotes the function that returns the popularity values of music track obtained from Spotify API and $p_{\mathrm{highest}}$ denotes the music track with the highest popularity value in the database.
**Novelty Reward**
  Many music-savvy users may want to focus on the latest music. Notably, Shih et al. argues that it is important to consider the novelty of music tracks in the playlist [42]. We design the reward based on the year in which music tracks were released so that the generated playlist contains more new music tracks. The novelty reward $R_{t}^{\mathrm{new}}$ is defined as follows:
  (15) $$R_{t}^{\mathrm{new}}=\frac{1}{N_{p}}\sum_{i=1}^{N_{p}}\frac{\mathrm{Year}\left(p_{t}^{i}\right)-\mathrm{Year}(m_{\mathrm{oldest}})}{\mathrm{Year}(m_{\mathrm{newest}})-\mathrm{Year}(m_{\mathrm{oldest}})},$$
  where Year(·) denotes the function that returns the year of the music track, $m_{\mathrm{oldest}}$ and $m_{\mathrm{newest}}$ denote the oldest and the newest music tracks in the database, respectively.

#### 4.4.3. Final Reward Function

Based on the above discussion, we present the final reward function. The ability to control the balance of the influence of each reward based on explicit inputs γ from the target user is a critical feature of our model. The explicit inputs γ are the four parameters for each reward, which are as follows:

(16) $$\gamma =\{\gamma_{\mathrm{pre}},\gamma_{\mathrm{aco}},\gamma_{\mathrm{pop}},\gamma_{\mathrm{new}}\},$$

where, $\gamma_{\mathrm{pre}}$, $\gamma_{\mathrm{aco}}$, $\gamma_{\mathrm{pop}}$ and $\gamma_{\mathrm{new}}$ denote the coefficient parameters of $R_{t}^{\mathrm{pre}}$, $R_{t}^{\mathrm{aco}}$, $R_{t}^{\mathrm{pop}}$ and $R_{t}^{\mathrm{new}}$, respectively.

However, because the above rewards have different distributions, we cannot make equal comparisons using a simple linear combination. To make equal comparisons between each reward, we introduce the empirical distribution function [55]. The empirical distribution function is one of the distribution functions in the field of statistics that can be defined as a step function that increases by 1/n for every *n* data point. We create new reward distributions $R_{t}^{\prime \mathrm{pre}}$, $R_{t}^{\prime \mathrm{aco}}$, $R_{t}^{\prime \mathrm{pop}}$ and $R_{t}^{\prime \mathrm{new}}$ that are robust to different variances and outliers using the empirical distribution function for distributing $R_{t}^{\mathrm{pre}}$, $R_{t}^{\mathrm{aco}}$, $R_{t}^{\mathrm{pop}}$ and $R_{t}^{\mathrm{new}}$,.

Finally, we define the final reward function based on the obtained new reward distribution as follows:

(17) $$\begin{array}{c} R_{t}=\gamma_{\mathrm{pre}}R_{t}^{\prime \mathrm{pre}}+\gamma_{\mathrm{aco}}R_{t}^{\prime \mathrm{aco}}+\gamma_{\mathrm{pop}}R_{t}^{\prime \mathrm{pop}}+\gamma_{\mathrm{new}}R_{t}^{\prime \mathrm{new}}. \end{array}$$

### 4.5. Optimization

In this subsection, we describe the optimization of our model. To obtain the optimal strategy based on the designed state, action, and reward, we trained the MDP using the REINFORCE [28], which is a standard policy gradient algorithm. Furthermore, we simultaneously trained the induction network to acquire future states to obtain better music prediction performance. The details are shown below.

#### 4.5.1. Training with Policy Gradient

In our music playlist generation task, we obtain a stochastic policy π that maximizes the expected cumulative reward J(Θ) for all target users. The derivative of J(Θ) can be obtained as follows:

(18) $$\nabla J\left(\Theta \right)=E_{\pi }\left[\sum_{U}\sum_{t^{\prime }=t}^{T}\gamma_{\pi }^{t^{\prime }-t}R_{t^{\prime }}\frac{\nabla \pi (a_{t}|s_{t};\Theta )}{\pi (a_{t}|s_{t};\Theta )}\right],$$

where $E{\left[\cdot \right]}_{\pi }$ denotes the expected value when the agent acts according to the policy π, $\gamma_{\pi }$ denotes the discount rate, Θ denotes all related parameters to learn, and T denotes the terminal time step of one episode. We use the REINFORCE [28] strategy to learn the parameters of the policy function. The agent generates D playlists every one time step. Specifically, for each state $s_{t}$, our model sample playlists $p^{1:N_{p}}\left(d\right)$ (1 ≤ d ≤ D) composed of $N_{p}$ music tracks according to policy function Equation (3). Given the playlist $p^{1:N_{p}}\left(d\right)$, the learning process is written as follows:

(19) $$\nabla \Theta =\sum_{t^{\prime }=t}^{T}\gamma_{\pi }^{t^{\prime }-t}R_{t^{\prime }}\frac{\nabla \pi (p_{t}^{1:N_{p}}\left(d\right)|s_{t};\Theta )}{\pi (p_{t}^{1:N_{p}}\left(d\right)|s_{t};\Theta )}.$$

To estimate a better cumulative reward, our model repeats the above process D times.

#### 4.5.2. Training the Induction Network

The key component of our model for predicting target users’ preferences is the induction network in Equation (9). To train such a neural network, a simple solution is to apply regression losses such as mean squared error (MSE). However, in our task, it is difficult to efficiently train an induction network using simple regression loss because of the sparse GT listening history. We then use the pairwise ranking strategy to train the induction network based on the idea of literature [25].

Based on the generated playlist, we can derive their music feature-based future preference representations based on Equation (9), which is denoted by $f_{t}^{\left(1\right)}$, $f_{t}^{\left(2\right)},\ldots ,f_{t}^{\left(D\right)}$. Our objective is to train the induction network so that accurate future preference representations can be acquired. To achieve this objective, we introduce pairwise comparisons as additional constraints to the induction network. Specifically, given $f_{t}^{\left(d\right)}$ and $f_{t}^{\left(d^{\prime }\right)}$, we first exploit the reward to determine the preference order over D playlists. Furthermore, we add pairwise constraints to train the induction network, where MLP($f_{t}^{\left(d\right)}$) > MLP($f_{t}^{\left(d^{\prime }\right)}$) if $R_{\mathrm{pre}}$($l_{t+1:N_{p}}^{u_{v}}$, $p_{t}^{1:N_{p}}\left(d\right)$) > $R_{\mathrm{pre}}$($l_{t+1:N_{p}}^{u_{v}}$, $p_{t}^{1:N_{p}}\left(d^{\prime }\right)$) for 1 ≤ d, $d^{\prime }$ ≤ D.

Finally, our model generates the playlist of $N_{p}$ music tracks for the target user based on the trained induction network and policy function. In the test phase, we first obtain the states and rewards corresponding to the target user’s listening history and the explicit inputs with the trained induction network. Based on the obtained states and rewards, the trained policy function outputs the playlist for the target user.

## 5. Experiment

In this section, we present the experiment that was used to confirm the effectiveness of our model by comparing it with conventional playlist generation and music recommendation methods. Our experiment evaluates our model in the following two aspects: (1) whether it can predict target users’ preferences based on their listening histories and (2) whether it can guide target users to appropriately new types of music tracks based on their explicit inputs. We present the details of the experimental settings in Section 5.1, and their results and discussion in Section 5.2.

### 5.1. Experimental Setting

In the experiment, we used 1006 users’ listening histories provided by the Spotify Million Playlist Dataset [56] and the metadata of music tracks provided by Spotify API. The dataset contains 57,880 music tracks, 1006 users, 14,973 artists, and 2517 genres of artists. Each artist belonged to at least one genre. The number of dimensions of the music feature $m_{i}$ obtained from TransE was set to 50. For the acoustic features of the music tracks, we used eight-dimensional values of danceability, energy, speeches, acoustics, valence, instrumentals, liveness, and tempo provided by the Spotify API. The popularity values of music tracks range from 0 to 100. In our dataset, the highest popularity of $p_{\mathrm{highest}}$ is 90, the year of $m_{\mathrm{oldest}}$ is 1935 and the year of $m_{\mathrm{newest}}$ is 2022. The training and test data are the first 90% and the last 10% of music tracks in each playlist, respectively. The number of music tracks in the generated playlist is set to 10.

To confirm the performance of the proposed method (PM), we compared the PM with the following four music playlist generation methods based on the basic RNN model, the graph exploration-based model, and the state-of-the-art KG- and RL-based recommendation models.

**CM1:**
  The method is based on GRU [53] trained by only the target users’ listening history
**CM2 [17]:**
  The playlist generation method is based on the exploration of the graph constructed from the acoustic similarities of the music tracks
**CM3 [49]:**
  The method is based on a deep RL-based music recommendation model that uses the KG constructed from users’ listening history and acoustic features of music tracks
**CM4 [36]:**
  The method is based on the item recommendation model and uses RL-based KG reasoning to explain recommendation results

The music playlist generation is a special case of music recommendation, as described in Section 2.1. Therefore, we used the state-of-the-art RL- and KG-based music recommendation model [17] and the item recommendation model [36], which is the predecessor of [17], as the comparison methods. Note that CM4 generates playlists in which the acoustic features of consecutive tracks are highly similar, and does not focus on prediction performance based on the target user’s listening history.

To make a very legitimate and reliable comparison of PMs and CMs, it is desirable to conduct a large-scale online evaluation where target users assess the quality of the generated playlist. However, it requires a complex infrastructure that is beyond the scope of this study. Instead of the large-scale online evaluation, we conducted a standard offline evaluation as performed in [42] using models based on real users. In the evaluation, we used seven PM-based models with different parameters of the reward function $R_{t}$, assuming target users with various backgrounds and unique preferences. The PM-based models and every parameter are shown in Table 1. For example, PM-A, PM-PN, and PM-ALL are models assuming a user who desires a playlist consisting of music tracks with similar acoustic features, a user who enjoys new and popular music tracks, and a user who focuses on all of the similarities of acoustic features of music tracks, novelty, and popularity of music tracks. Since the parameter γ is user-controllable, in practice, the target user can sequentially generate user-specific playlists by setting the desired gamma value. Naturally, the target users can also set γ values other than these seven model’s γ values. Note that $\gamma_{\mathrm{pre}}$ is not set to 0 because the state cannot be updated if the induction network receives vacant value of $R_{\mathrm{pre}}$.

To evaluate aspect (1), we use normalized discounted cumulative gain@k (nDCG@k) and Hit Rate@k (k = 1, 5, 10), which are commonly used to evaluate the prediction performance of the playlist generation [57,58]. However, to objectively evaluate aspect (2), we used the following three evaluation metrics corresponding to the rewards $R_{\mathrm{aco}}^{\prime }$, $R_{\mathrm{pop}}^{\prime }$ and $R_{\mathrm{new}}^{\prime }$.

**Acoustic Similarity Metric** $M_{\mathrm{aco}}$

  To measure the similarity of acoustic features of successive music tracks in the generated playlist, we designed $M_{\mathrm{aco}}$ as follows:
  (20) $$M_{\mathrm{aco}}=\sum_{U}\frac{1}{N_{u_{v}}^{p}}\sum_{i=0}^{N_{u_{v}}^{p}-1}\frac{{p^{i}}_{u_{v},\mathrm{aco}}\cdot {p_{u_{v},\mathrm{aco}}^{i+1}}^{⊤}}{\left|\right|{p^{i}}_{u_{v},\mathrm{aco}}\left|\right|\cdot \left|\right|{p_{u_{v},\mathrm{aco}}^{i+1}}^{⊤}\left|\right|},$$
  where $N_{u_{v}}^{p}$ denotes the number of music tracks in the playlist generated for the target user $u_{v}$, and ${p^{i}}_{u_{v},\mathrm{aco}}$ denotes the acoustic feature of i-th music track in the playlist generated for the target user $u_{v}$.
**Popularity Metric** $M_{\mathrm{pop}}$

  To evaluate whether the generated playlist consists of music tracks with high popularity, we used the average values as the popularity metric. The popularity metric $M_{\mathrm{pop}}$ is defined as follows:
  (21) $$M_{\mathrm{pop}}=\sum_{U}\frac{1}{N_{u_{v}}^{p}}\sum_{i=1}^{N_{u_{v}}^{p}}\mathrm{Popularity}(p_{u_{v}}^{i}),$$
  where $p_{u_{v}}^{i}$ denotes the music track in the playlist generated for the target user $u_{v}$.
**Novelty Metric** $M_{\mathrm{new}}$

  To evaluate whether new music tracks are included in the generated playlist, we used the average of the year that the music tracks were released as a novelty metric. The novelty metric $M_{\mathrm{new}}$ is defined as follows:
  (22) $$M_{\mathrm{new}}=\sum_{U}\frac{1}{N_{u_{v}}^{p}}\sum_{i=1}^{N_{u_{v}}^{p}}\mathrm{Year}(p_{u_{v}}^{i}).$$

### 5.2. Results and Discussion

In this subsection, we present and discuss the results of our experiment. First, the values of nDCG@k and Hit Rate@k for the PM-based models and CMs are shown in Table 2. It shows that all PM-based models achieve higher values of nDCG@k and Hit Rate@k than all CMs for all values of k. PM outperforms CM1, indicating the effectiveness of introducing RL to the music playlist generation for analyzing not only the target user’s short-term preference but also the long-term preference. For CM3 and CM4, we use the target user’s listening history as a component of the KG, not as a sequence. PM can capture the target user’s short-term and long-term preference using the target user’s listening history as a sequence and it can be analyzed on the basis of the GRU and RL. The prediction performance of CM2 is not high because it only considers the similarity of acoustic features of the music tracks. Furthermore, we compare PM-based models with each other. PM-P and PM-N are the PM-based models with the highest values of nDCG@1, nDCG@10 and Hit Rate@k (k = 1, 5, 10), and the highest values of nDCG@5, respectively. The prediction parameter $\gamma_{\mathrm{pre}}$ of PM-P and PM-N is 0.5, which is greater than that of other models. This implies that the similarity of music features obtained from KG contributes to the improvement of the prediction performance. According to the above discussion, PM can accurately predict target users’ preferences using RL and KG.

Table 3 shows the values of $M_{\mathrm{aco}}$, $M_{\mathrm{pop}}$, and $M_{\mathrm{new}}$ for the PM-based models and CMs. For $M_{\mathrm{pop}}$ and $M_{\mathrm{new}}$, the models with the highest values are PM-P and PM-N, respectively, and the models with the second-largest values are PM-AP and PM-PN, respectively. These results imply that our model can generate playlists comprising popular and recent music for target users who desire popular and new music tracks. For $M_{\mathrm{aco}}$, the model with the largest value is CM2. This is not a surprising result because CM2 is a method that specializes only in making the similarity of acoustic features of successive music tracks higher. Table 3 shows that CM2 has poor predictive performance and cannot control $M_{\mathrm{pop}}$ and $M_{\mathrm{new}}$. The models with the second highest and third-highest values of $M_{\mathrm{aco}}$ are PM-A and PM-AN, respectively. This means that our model can also accommodate the target user’s specific preferences regarding the similarity of acoustic features of music tracks.

**Figure 2:**
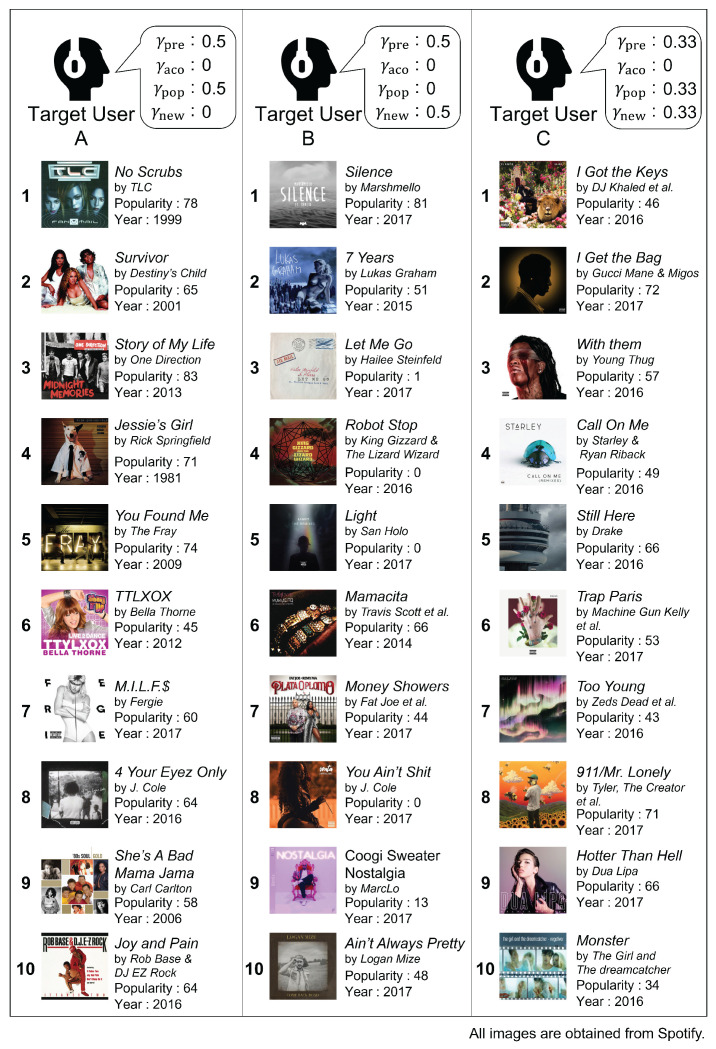
The examples of the generated playlists. The playlists generated for target users A and B contain many popular and new music tracks, respectively. Furthermore, our model generated a playlist consisting of both many popular and new music tracks for the target user C.

Figure 2 shows examples of the generated playlists. According to the playlist examples, it is possible to generate playlists that meet the target users’ requirements by adjusting the parameters of the reward function. Therefore, we conclude that our model can guide target users to music tracks that can satisfy their unique preferences.

## 6. Conclusions

In this study, we present a controllable music playlist generation method based on a knowledge graph and reinforcement learning. Our model can predict target users’ preferences based on their listening histories and guide target users to new types of music tracks based on their explicit inputs. The experimental results show that the proposed method outperforms existing playlist generation methods.

However, our model remains the three following problems. The first problem is that the effectiveness of PM has not been verified by actual users. In the experiment, we evaluate the effectiveness of PM by using seven models assuming a user, however, the practicality of PM based on actual user interactions and feedback has not been evaluated. Therefore, we will verify the effectiveness of PM through a large-scale online evaluation in future work. The second problem is that the reward function is incomplete. We introduced four indices into the reward function, however, we should also consider other various factors, such as the artist and label of the music. In future work, we will set the reward function that better responds to users’ niche preferences by introducing additional indices. Furthermore, we will estimate the parameters of the reward function appropriate for each target user. The third problem is that PM did not consider the structure and length of music tracks. If the generated playlist consists of long music tracks, target users may become bored due to the redundancy of the playlist. In addition, the position of the chorus and the length of the intro may also affect user satisfaction. In future work, we will take into account the relationship between the structure of music tracks and playlist quality.

## Figures and Tables

**Table 1:** PM-based models and each parameter value of the reward function $R_{t}$.

Parameter	$\gamma_{\mathrm{pre}}$	$\gamma_{\mathrm{aco}}$	$\gamma_{\mathrm{pop}}$	$\gamma_{\mathrm{new}}$
PM-A	0.5	0.5	0	0
PM-P	0.5	0	0.5	0
PM-N	0.5	0	0	0.5
PM-AP	0.33	0.33	0.33	0
PM-AN	0.33	0.33	0	0.33
PM-PN	0.33	0	0.33	0.33
PM-ALL	0.25	0.25	0.25	0.25

**Table 2:** The values of nDCG@*k* and Hit Rate@*k* (*k* = 1, 5, 10). The bold values are the maximum values for each evaluation metrics, respectively.

Metric		nDCG@*k* [$\times 10^{-2}$]			Hit Rate@*k* [%]	
	*k* = 1	*k* = 5	*k* = 10	*k* = 1	*k* = 5	*k* = 10
PM-A	18.2	24.7	26.2	18.2	30.9	37.9
PM-P	**19.3**	25.6	**27.9**	**19.3**	**33.4**	**40.4**
PM-N	18.2	**26.1**	26.8	18.2	32.9	39.6
PM-AP	17.5	23.5	24.8	17.5	29.8	36.8
PM-AN	17.0	24.9	24.5	17.0	27.2	34.2
PM-PN	18.7	22.1	24.6	18.7	30.5	38.1
PM-ALL	18.0	23.0	24.1	18.0	29.0	36.0
CM1	10.2	13.9	17.2	10.2	21.8	28.8
CM2 [17]	0.00	0.52	0.40	0.00	0.43	1.03
CM3 [49]	3.42	5.30	6.22	3.42	6.71	11.5
CM4 [36]	3.59	4.30	5.94	3.59	7.05	12.0

**Table 3:** The values of $M_{\mathrm{aco}}$, $M_{\mathrm{pop}}$, and $M_{\mathrm{new}}$. The bold and underlined values are the maximum and second highest values for each evaluation metrics, respectively.

Metrics	$M_{\mathrm{aco}}$	$M_{\mathrm{pop}}$	$M_{\mathrm{new}}$
PM-A	0.90	19.0	2001.9
PM-P	0.57	**52.8**	2012.4
PM-N	0.64	37.6	**2013.4**
PM-AP	0.81	50.6	2006.6
PM-AN	0.86	27.2	2011.4
PM-PN	0.65	48.0	2012.8
PM-ALL	0.73	42.0	2009.8
CM1	0.56	41.2	2005.4
CM2 [17]	**0.97**	19.3	2004.2
CM3 [49]	0.65	38.6	2006.6
CM4 [36]	0.60	40.7	2007.0

## Data Availability

Publicly available datasets were analyzed in this study. This data can be found here: https://www.aicrowd.com/challenges/spotify-million-playlist-dataset-challenge (accessed on 12 March 2022).
